# Bis[2,6-bis­(1*H*-benzimidazol-2-yl)pyridine]ruthenium(II) bis(hexa­fluorido­phosphate) diethyl ether tris­olvate

**DOI:** 10.1107/S2414314624002694

**Published:** 2024-03-28

**Authors:** Layla M. Althubyani, Brian J. MacLean, Katherine N. Robertson, Manuel A.S. Aquino

**Affiliations:** a Department of Chemistry, St. Francis Xavier University, Antigonish, Nova Scotia, Canada, B2G 2W5; b Department of Chemistry, Saint Mary’s University, Halifax, Nova Scotia, Canada, B3H 3C3; Goethe-Universität Frankfurt, Germany

**Keywords:** crystal structure, ruthenium(II) complex, Bimpy ligand

## Abstract

The title compound, [Ru(C_19_H_13_N_5_)_2_](PF_6_)_2_·3C_4_H_10_O, was obtained from the reaction of Ru(bimpy)Cl_3_ [bimpy is 2,6-bis­(1*H*-benzimidazol-2-yl)pyridine] and bimpy in refluxing ethanol followed by recrystallization from diethyl ether/aceto­nitrile. At 125 K the complex has ortho­rhom­bic (*Pca*2_1_) symmetry. It is remarkable that the structure is almost centrosymmetric. However, refinement in space group *Pbcn* leads to disorder and definitely worse results. It is of inter­est with respect to potential catalytic reduction of CO_2_. The structure displays N—H⋯O, N—H⋯F hydrogen bonding and significant π–π stacking and C—H⋯π stacking inter­actions.

## Abstract 2

The cationic complex of the title salt, [Ru(C_19_H_13_N_5_)_2_](PF_6_)_2_·3C_4_H_10_O, has the Ru atom in a slightly distorted octa­hedral environment of two tridentate benzimdazolyl-pyridine ligands and displays extensive π–π and C—H⋯π inter­actions.

## Graphical abstract

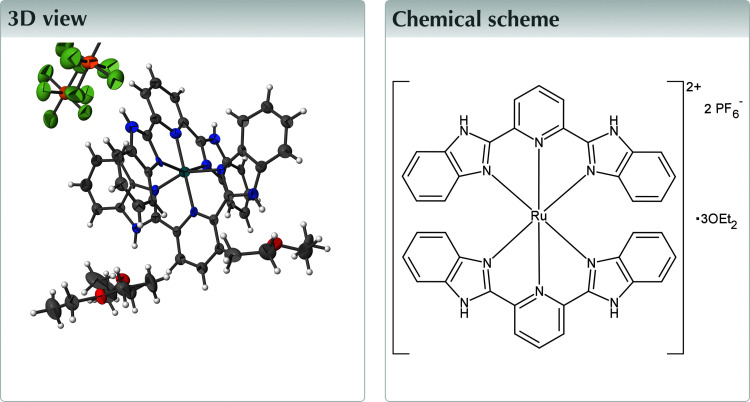

## Structure description

Ruthenium(II) complexes that contain polypyridine ligands enjoy enormous popularity in the research community because of their inter­esting photochemical, electrochemical, and catalytic properties (Juris *et al.*, 1988 ▸). Similar to what is found in 2,2′:6′,2′′ terpyridine, the tri­imine structure, 2,6-bis(1*H*-benzimidazol-2-yl)pyridine (bimpy), offers a tridentate pocket for its coordination complexes; however, the imidazole units present a more convenient opportunity for tuning the electronics of donor–acceptor inter­actions (Groff *et al.*, 2023 ▸). Our inter­est in bimpy complexes of ruthenium stems from reports of their activity in mediation of CO_2_ by electrochemical reduction (Chen *et al.*, 2011 ▸). This is the first crystal structure of a bis-bimpy complex of Ru^II^ that we are aware of.

**Figure 1:**
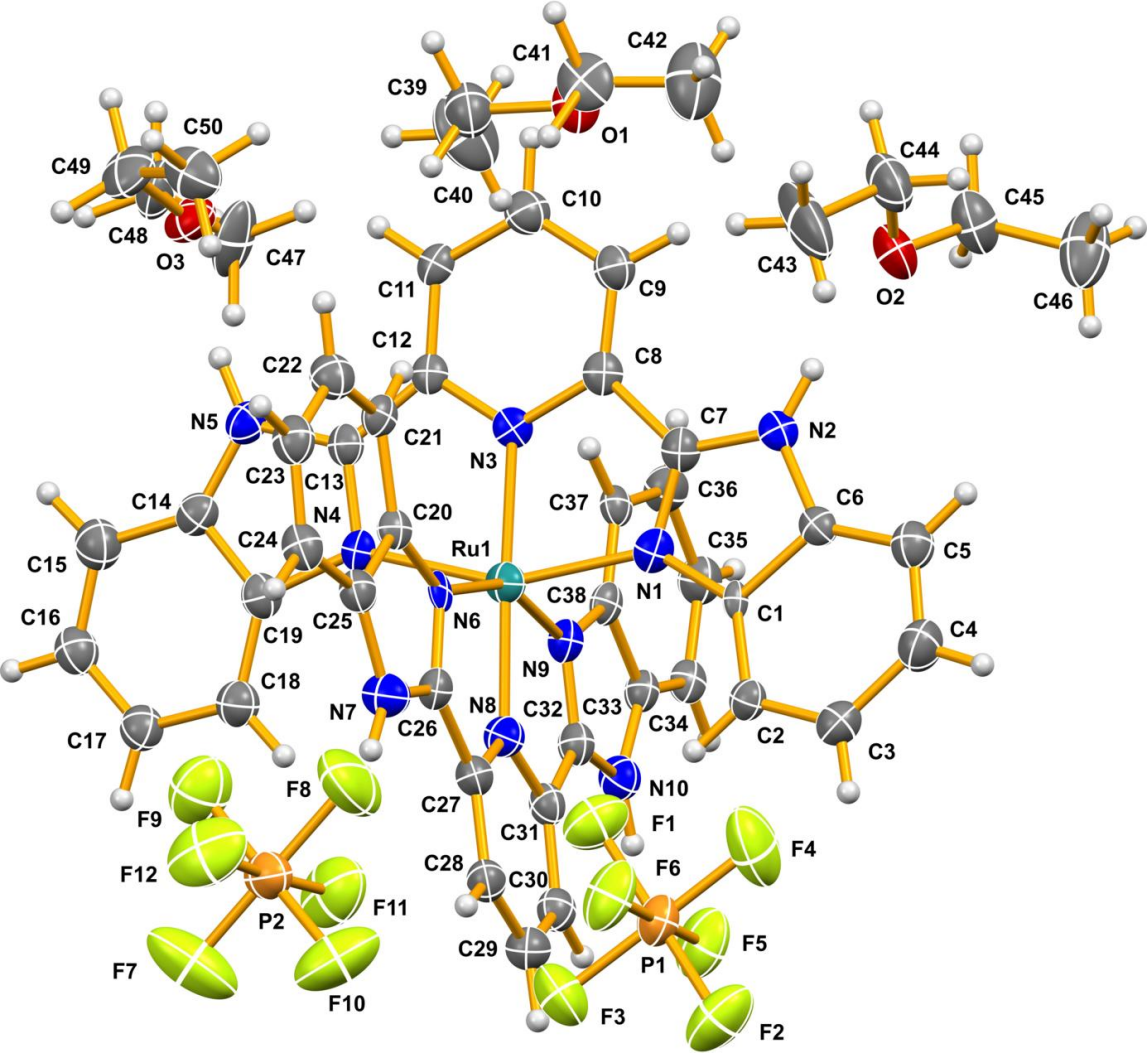
The structures of the molecular entities of the title compound with displacement ellipsoids at the 50% probability level.

**Figure 2:**
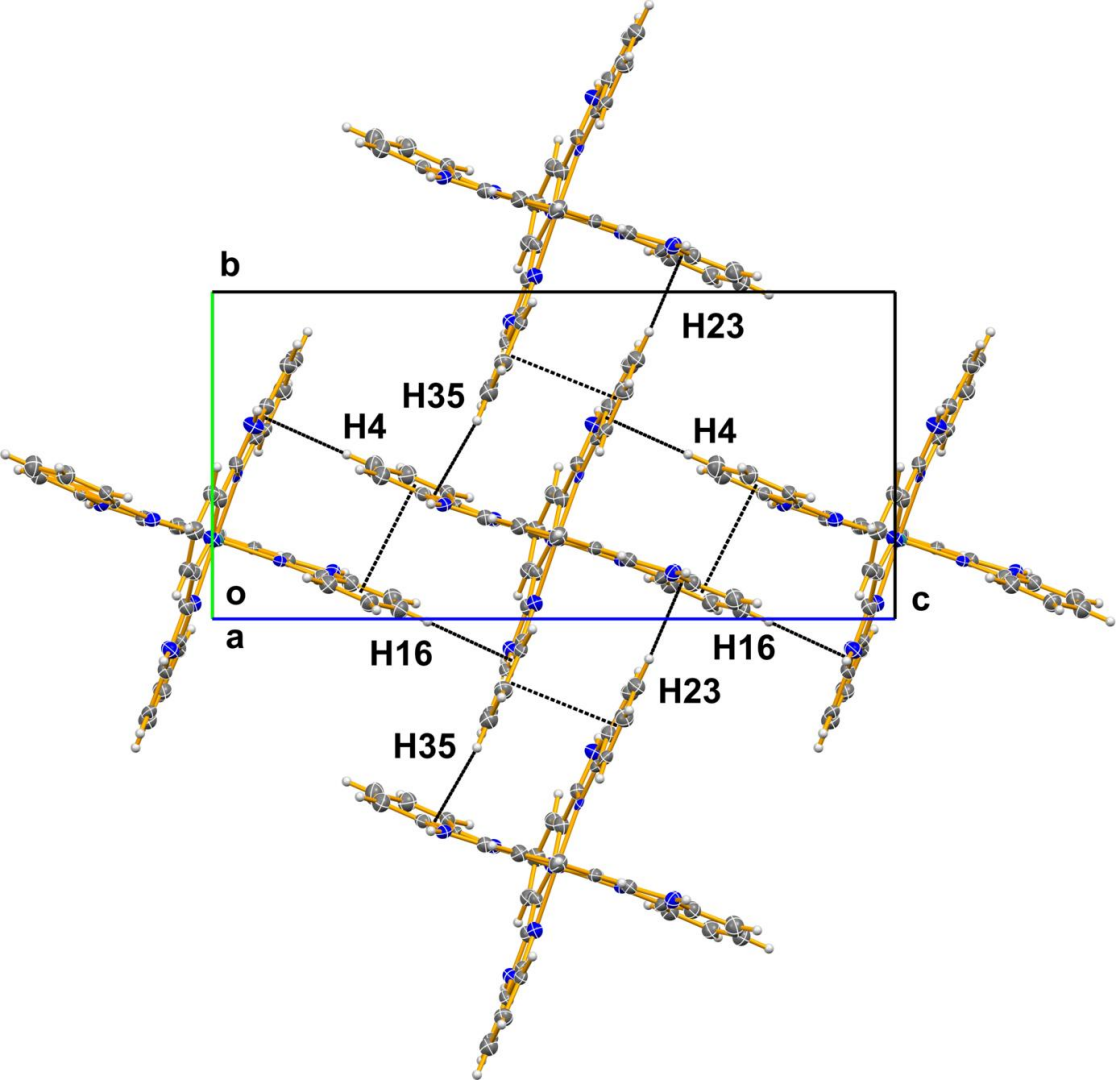
Packing diagram viewed along [100] showing both the C—H⋯π and π–π inter­actions (dashed lines).

The solvated title salt consists of the complex cation, ruthenium(II) bis­(bimpy), two hexa­fluorido­phosphate anions and three diethyl ether mol­ecules of solvation (Fig. 1 ▸). The two tris-chelating bimpy ligands both coordinate through three of their nitro­gen atoms to the central Ru^II^ atom, perpendicular to each other in a meridional fashion, forming a slightly distorted octa­hedral environment. As a result of the *Pca*2_1_ space group, all of the Ru—N bonds are unique. The two Ru^II^—N(pyridin­yl) bond lengths are: Ru1—N8 = 1.983 (9) Å and Ru1—N3 = 2.011 (8) Å and the four Ru(II)—N(benzimidazol­yl) bonds, Ru1—N1, Ru1—N4, Ru1—N6 and Ru1—N9 are slightly longer and range from 2.046 (13) to 2.104 (12) Å. These same bond lengths in [Ru(bimpy)(trpy)](ClO_4_)_2_ (where trpy = 2,2′:6′,2"-terpyridine) are 2.017 (7) Å and 2.067 (7)–2.072 (7) Å, respectively (Singh *et al.*, 2008 ▸). While the complex does show hydrogen bonding between the imidazolyl N—H groups and the two hexa­fluorido­phosphate anions and two of the three diethyl ether solvate mol­ecules (Table 1 ▸), more inter­esting are the π–π stacking and C—H⋯π inter­actions (Fig. 2 ▸). The shortest π–π inter­actions are between the six-membered (benzene) rings of adjacent benzimidazolyl ligands and range from 3.639 (9) to 3.675 (8) Å. The C—H⋯π inter­actions involve a C—H group on these same benzene ring portions of the benzimidazolyl and adjacent benzimidazolyl benzene rings and have carbon to π-ring distances ranging from 3.487 (16) to 3.792 (18) Å.

## Synthesis and crystallization

[Ru(bimpy)_2_](PF_6_)_2_ was synthesized through reaction of Ru(bimpy)Cl_3_ (Yu *et al.*, 1999 ▸) with bimpy (Xu *et al.*, 2007 ▸). Bimpy (0.0646 g, 0.21 mmol) and Ru(bimpy)Cl_3_ (0.1064 g, 0.21 mmol) were added to a warm solution of aqueous ethanol (75%_vol_). Tri­ethyl­amine (0.25 ml) was added to the mixture. The solution was refluxed under argon for 24 h, then cooled to room temperature. The insoluble materials were removed by filtration and the complex was precipitated by the addition of a saturated aqueous solution of NH_4_PF_6_ while cooling at 4°C overnight. The resulting, brown precipitate was filtered and washed with water, air-dried, and then washed with diethyl ether (3 × 10 ml). Crystals of [Ru(bimpy)_2_](PF_6_)_2_ were grown by slow diffusion of diethyl ether into an aceto­nitrile solution of the product, yielding dark-red crystals (0.1555 g, 75%). ^1^H NMR (400 MHz, DMSO-*d*_6_) (p.p.m.): 15.01 (s, 4*H*), 8.89 (*d*, *J* = 7.9 Hz, 4H), 8.77 (*t*, *J* = 7.9 Hz, 2H), 7.59 (*d*, *J* = 8.1 Hz, 4H), 7.25 (*dd*, *J* = 7.6 Hz, 4H), 7.02 (*dd*, *J* =7.6 Hz, 4H), 6.03 (*d*, *J* = 8.125 Hz, 4H). ^13^C NMR (400 MHz, DMSO-*d*_6_) (p.p.m.): 151.51, 149.78, 140.83, 136.70, 133.34, 125.54, 124.69, 122.16, 114.50, 114.28. IR (KBr) (cm^−1^): 3364 (*br*), 1613 (*w*), 1597 (*w*), 1487 (*w*), 1458 (*w*), 1384 (*w*), 1322, 1297, 1233 (*w*), 1149 (*w*), 1021 (*w*), 851 (s), 760 (*w*), 743 (*s*). ESI–MS: *m*/*z* calculated for C_38_ H_26_ N_10_ Ru (*M* ^2+^): 362.0693, found: 362.0676.

## Refinement

Crystal data, data collection and structure refinement details are summarized in Table 2 ▸. The data were first integrated to a resolution of 0.75 Å but during the final refinement, the data were cut at a resolution of 0.80 Å (*θ*_max_ = 26.37°) using a *SHEL* instruction to remove some of the noise. The unit cell was determined to be ortho­rhom­bic and it was found that the structure could be refined in either the centrosymmetric space group *Pbcn* or in the non-centrosymmetric space group *Pca*2_1_. Ultimately, the non-centrosymmetric space group was chosen, giving an asymmetric unit that contained one complete cation and two complete PF_6_ anions. The crystal was also found to be solvated, containing three complete mol­ecules of diethyl ether in the asymmetric unit. The *Pca*2_1_ refinement had many atoms that had a tendency to become non-positive definite during the refinement, presumably because it was so close to being centrosymmetric. As a result, the displacement parameters of most atoms were restrained to be more isotropic during the refinement using global ISOR restraints. In addition, a rigid bond restraint was placed over all of the heavy atoms in the structure. The structure was treated as an inversion twin with the BASF parameter refining to 0.45 (12). The error is too large to say if this is different from the 0.50 expected for a centrosymmetric structure but it is possible to say it is not 0 or 1 (expected for a non-twinned non-centrosymmetric structure).

Initial *E* statistics suggested that the correct space group was non-centrosymmetric and the best solution in *SHELXT* was also in a non-centrosymmetric space group. The structure was thus first refined in the non-centrosymmetric space group *Pca*2_1_. In this space group the final *R*-factor was high [*R*(reflections) = 0.0920 (9404) and *wR*2(reflections) = 0.1987 (11121)] and there was a level B *checkCIF* alert that the precision of the C—C bonds was low. A level G *checkCIF* alert suggested that there was an 89% fit to a centrosymmetric structure and that the alternative space group *Pbcn* should be used. Refinement in this space group was then carried out, but with much worse results overall. In the centrosymmetric space group, the central ring of the cation and the solvent mol­ecules were all disordered (12% disorder in total). The statistics of the final refinement carried out under similar conditions to the non-centrosymmetric case were also much higher [*R*(reflections) = 0.1494 (5022) and *wR*2(reflections) = 0.3162 (5578)]. For these reasons, refinement in the non-centrosymmetric space group was chosen and the *Pca*2_1_ results are presented here.

## Supplementary Material

Crystal structure: contains datablock(s) I. DOI: 10.1107/S2414314624002694/bt4148sup1.cif

Structure factors: contains datablock(s) I. DOI: 10.1107/S2414314624002694/bt4148Isup2.hkl

CCDC reference: 2343078

Additional supporting information:  crystallographic information; 3D view; checkCIF report

## Figures and Tables

**Table 1:** Hydrogen-bond geometry (Å, °)

*D*—H⋯*A*	*D*—H	H⋯*A*	*D*⋯*A*	*D*—H⋯*A*
N2—H2*N*⋯O2	0.90 (3)	1.85 (5)	2.730 (18)	166 (17)
N5—H5*N*⋯O3	0.91 (3)	1.81 (5)	2.704 (19)	170 (18)
N7—H7*N*⋯F8	0.90 (3)	2.58 (7)	3.30 (2)	137 (8)
N7—H7*N*⋯F11	0.90 (3)	2.05 (4)	2.93 (2)	167 (10)
N10—H10*N*⋯F6^i^	0.89 (3)	2.16 (3)	3.028 (19)	166 (6)

Symmetry code: (i) 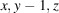 .

**Table 2:** Experimental details

Crystal data	
Chemical formula	[Ru(C_19_H_13_N_5_)_2_](PF_6_)_2_·3C_4_H_10_O
*M*_r_	1236.05
Crystal system, space group	Orthorhombic, *P* *c* *a*2_1_
Temperature (K)	125
*a*, *b*, *c* (Å)	26.718 (4), 9.8834 (13), 20.648 (3)
*V* (Å^3^)	5452.4 (12)
*Z*	4
Radiation type	Mo *K*α
μ (mm^−1^)	0.44
Crystal size (mm)	0.26 × 0.18 × 0.12

Data collection	
Diffractometer	Bruker APEXII CCD
Absorption correction	Multi-scan (*SADABS*; Krause *et al.*, 2015 ▸)
*T*_min_, *T*_max_	0.027, 0.049
No. of measured, independent and observed [*I* > 2σ(*I*)] reflections	54516, 11121, 9404
*R*_int_	0.058
(sin θ/λ)_max_ (Å^−1^)	0.625

Refinement	
*R*[*F* ^2^ > 2σ(*F* ^2^)], *wR*(*F* ^2^), *S*	0.092, 0.199, 1.18
No. of reflections	11121
No. of parameters	716
No. of restraints	1049
H-atom treatment	H atoms treated by a mixture of independent and constrained refinement
Δρ_max_, Δρ_min_ (e Å^−3^)	2.08, −1.44
Absolute structure	Refined as an inversion twin
Absolute structure parameter	0.45 (12)

Computer programs: *APEX2* and *SAINT* (Bruker, 2009 ▸), *SHELXT2014* (Sheldrick, 2015*a* ▸), *SHELXL2018/3* (Sheldrick, 2015*b* ▸) and *Mercury* (Macrae *et al.*, 2020 ▸).

## full crystallographic data

### Crystal data

**Table:**

[Ru(C_19_H_13_N_5_)_2_](PF_6_)_2_·3C_4_H_10_O	*D*_x_ = 1.506 Mg m^−^^3^
*M**_r_* = 1236.05	Mo *K*α radiation, λ = 0.71073 Å
Orthorhombic, *P**c**a*2_1_	Cell parameters from 9996 reflections
*a* = 26.718 (4) Å	θ = 2.2–28.3°
*b* = 9.8834 (13) Å	µ = 0.44 mm^−^^1^
*c* = 20.648 (3) Å	*T* = 125 K
*V* = 5452.4 (12) Å^3^	Rectangular prism, dark brown
*Z* = 4	0.26 × 0.18 × 0.12 mm
*F*(000) = 2528	

### Data collection

**Table:**

Bruker APEXII CCD diffractometer	11121 independent reflections
Radiation source: sealed tube	9404 reflections with *I* > 2σ(*I*)
Graphite monochromator	*R*_int_ = 0.058
φ and ω scans	θ_max_ = 26.4°, θ_min_ = 2.1°
Absorption correction: multi-scan (SADABS; Krause *et al.*, 2015)	*h* = −33→33
*T*_min_ = 0.027, *T*_max_ = 0.049	*k* = −12→12
54516 measured reflections	*l* = −25→25

### Refinement

**Table:**

Refinement on *F*^2^	Hydrogen site location: mixed
Least-squares matrix: full	H atoms treated by a mixture of independent and constrained refinement
*R*[*F*^2^ > 2σ(*F*^2^)] = 0.092	*w* = 1/[σ^2^(*F*_o_^2^) + 59.8553*P*] where *P* = (*F*_o_^2^ + 2*F*_c_^2^)/3
*wR*(*F*^2^) = 0.199	(Δ/σ)_max_ < 0.001
*S* = 1.18	Δρ_max_ = 2.08 e Å^−^^3^
11121 reflections	Δρ_min_ = −1.44 e Å^−^^3^
716 parameters	Absolute structure: Refined as an inversion twin
1049 restraints	Absolute structure parameter: 0.45 (12)
Primary atom site location: dual	

### Special details

**Table:**

Geometry. All esds (except the esd in the dihedral angle between two l.s. planes) are estimated using the full covariance matrix. The cell esds are taken into account individually in the estimation of esds in distances, angles and torsion angles; correlations between esds in cell parameters are only used when they are defined by crystal symmetry. An approximate (isotropic) treatment of cell esds is used for estimating esds involving l.s. planes.
Refinement. Refined as a 2-component inversion twin.

### Fractional atomic coordinates and isotropic or equivalent isotropic displacement parameters (Å^2^)

**Table:**

	*x*	*y*	*z*	*U*_iso_*/*U*_eq_	
Ru1	0.24603 (3)	0.24442 (12)	0.50446 (12)	0.0272 (2)	
P1	0.4281 (2)	0.6827 (5)	0.3798 (2)	0.0392 (11)	
P2	0.4255 (2)	0.8396 (6)	0.6165 (2)	0.0438 (12)	
F1	0.3806 (5)	0.6486 (12)	0.4238 (6)	0.060 (3)	
F2	0.4753 (6)	0.7193 (15)	0.3363 (7)	0.075 (4)	
F3	0.4646 (5)	0.6473 (13)	0.4380 (6)	0.064 (3)	
F4	0.3917 (6)	0.7185 (16)	0.3226 (6)	0.081 (4)	
F5	0.4295 (5)	0.5311 (12)	0.3571 (6)	0.063 (3)	
F6	0.4258 (5)	0.8365 (11)	0.4050 (5)	0.060 (3)	
F7	0.4672 (6)	0.7920 (16)	0.6616 (9)	0.099 (5)	
F8	0.3811 (6)	0.8839 (19)	0.5686 (7)	0.098 (5)	
F9	0.3833 (6)	0.7858 (15)	0.6640 (6)	0.074 (4)	
F10	0.4666 (6)	0.8928 (15)	0.5686 (8)	0.088 (4)	
F11	0.4239 (5)	0.6940 (14)	0.5810 (7)	0.080 (4)	
F12	0.4239 (6)	0.9832 (14)	0.6496 (8)	0.083 (4)	
O1	0.0440 (5)	0.7937 (12)	0.4605 (6)	0.043 (3)	
O2	0.0846 (5)	0.3936 (13)	0.2722 (6)	0.048 (3)	
O3	0.0748 (5)	0.1501 (12)	0.7178 (6)	0.042 (3)	
N1	0.2297 (5)	0.3044 (12)	0.4120 (6)	0.028 (2)	
N2	0.1715 (5)	0.3491 (12)	0.3385 (6)	0.026 (2)	
H2N	0.141 (3)	0.352 (18)	0.320 (8)	0.039*	
N3	0.1708 (3)	0.2377 (10)	0.5067 (9)	0.0227 (17)	
N4	0.2307 (5)	0.1768 (11)	0.5990 (6)	0.021 (2)	
N5	0.1704 (6)	0.1409 (14)	0.6749 (6)	0.031 (3)	
H5N	0.140 (3)	0.140 (19)	0.694 (8)	0.046*	
N6	0.2623 (5)	0.4355 (11)	0.5373 (5)	0.020 (2)	
N7	0.3222 (5)	0.5914 (14)	0.5605 (7)	0.036 (3)	
H7N	0.3513 (13)	0.636 (9)	0.566 (8)	0.053*	
N8	0.3201 (3)	0.2446 (14)	0.4988 (9)	0.030 (2)	
N9	0.2618 (5)	0.0479 (13)	0.4726 (6)	0.029 (2)	
N10	0.3184 (5)	−0.0946 (14)	0.4384 (7)	0.032 (3)	
H10N	0.3482 (12)	−0.129 (7)	0.429 (8)	0.049*	
C1	0.2541 (5)	0.3511 (12)	0.3577 (6)	0.016 (2)	
C2	0.3034 (6)	0.3852 (15)	0.3447 (7)	0.026 (3)	
H2	0.328622	0.370813	0.376313	0.032*	
C3	0.3153 (7)	0.4402 (17)	0.2855 (8)	0.037 (3)	
H3	0.349201	0.461762	0.275920	0.044*	
C4	0.2769 (8)	0.465 (2)	0.2374 (9)	0.044 (4)	
H4	0.286593	0.502895	0.196940	0.053*	
C5	0.2275 (7)	0.4384 (17)	0.2473 (9)	0.037 (3)	
H5	0.202140	0.455960	0.216151	0.044*	
C6	0.2178 (6)	0.3816 (14)	0.3086 (7)	0.025 (3)	
C7	0.1808 (7)	0.3087 (16)	0.3992 (7)	0.029 (3)	
C8	0.1445 (6)	0.2777 (16)	0.4518 (7)	0.026 (3)	
C9	0.0937 (7)	0.2833 (18)	0.4488 (8)	0.032 (3)	
H9	0.076700	0.306661	0.410022	0.038*	
C10	0.0677 (4)	0.2528 (18)	0.5061 (12)	0.036 (2)	
H10	0.032208	0.257357	0.506813	0.043*	
C11	0.0934 (6)	0.2163 (16)	0.5614 (7)	0.025 (3)	
H11	0.075601	0.192158	0.599537	0.030*	
C12	0.1461 (6)	0.2152 (16)	0.5609 (7)	0.023 (2)	
C13	0.1806 (6)	0.1806 (14)	0.6111 (7)	0.025 (3)	
C14	0.2141 (7)	0.1101 (16)	0.7026 (8)	0.031 (3)	
C15	0.2286 (8)	0.0600 (18)	0.7645 (9)	0.043 (4)	
H15	0.204639	0.047661	0.797942	0.051*	
C16	0.2787 (7)	0.0296 (18)	0.7745 (8)	0.037 (3)	
H16	0.287942	−0.008088	0.815065	0.045*	
C17	0.3151 (7)	0.0501 (17)	0.7303 (8)	0.036 (3)	
H17	0.348683	0.026841	0.740472	0.043*	
C18	0.3039 (7)	0.1051 (17)	0.6699 (8)	0.034 (3)	
H18	0.328848	0.125378	0.638626	0.041*	
C19	0.2526 (6)	0.1284 (15)	0.6588 (7)	0.029 (3)	
C20	0.2400 (6)	0.5502 (14)	0.5638 (7)	0.022 (3)	
C21	0.1872 (6)	0.5763 (14)	0.5768 (6)	0.026 (3)	
H21	0.161950	0.511548	0.567312	0.031*	
C22	0.1762 (6)	0.6978 (16)	0.6032 (8)	0.036 (3)	
H22	0.142021	0.719526	0.610477	0.043*	
C23	0.2129 (6)	0.7943 (16)	0.6207 (7)	0.036 (3)	
H23	0.202184	0.876976	0.639669	0.043*	
C24	0.2631 (7)	0.7742 (18)	0.6115 (8)	0.033 (3)	
H24	0.287559	0.838158	0.624789	0.040*	
C25	0.2760 (6)	0.6488 (16)	0.5801 (8)	0.029 (3)	
C26	0.3113 (6)	0.4726 (14)	0.5348 (7)	0.026 (3)	
C27	0.3465 (5)	0.3682 (14)	0.5110 (8)	0.030 (3)	
C28	0.3973 (5)	0.3810 (14)	0.4988 (7)	0.030 (3)	
H28	0.414240	0.463642	0.506987	0.036*	
C29	0.4226 (5)	0.2700 (15)	0.4742 (7)	0.035 (3)	
H29	0.457377	0.275983	0.465126	0.042*	
C30	0.3968 (5)	0.1492 (15)	0.4628 (6)	0.030 (3)	
H30	0.414388	0.071711	0.447839	0.036*	
C31	0.3459 (6)	0.1425 (14)	0.4733 (7)	0.028 (3)	
C32	0.3105 (6)	0.0362 (15)	0.4618 (7)	0.029 (3)	
C33	0.2726 (6)	−0.1596 (15)	0.4308 (7)	0.026 (3)	
C34	0.2598 (6)	−0.2854 (16)	0.4076 (7)	0.027 (3)	
H34	0.283975	−0.350201	0.394395	0.032*	
C35	0.2095 (6)	−0.3095 (17)	0.4051 (8)	0.036 (3)	
H35	0.198913	−0.393808	0.387753	0.043*	
C36	0.1714 (6)	−0.2182 (16)	0.4265 (8)	0.035 (3)	
H36	0.136846	−0.240112	0.423405	0.042*	
C37	0.1876 (5)	−0.0952 (14)	0.4520 (6)	0.024 (3)	
H37	0.164107	−0.031994	0.468619	0.029*	
C38	0.2365 (6)	−0.0668 (14)	0.4530 (7)	0.024 (3)	
C39	0.0503 (10)	0.607 (2)	0.5342 (11)	0.075 (7)	
H39A	0.052672	0.582148	0.580088	0.113*	
H39B	0.020112	0.565792	0.515506	0.113*	
H39C	0.079855	0.572996	0.511162	0.113*	
C40	0.0475 (8)	0.7574 (19)	0.5278 (8)	0.048 (4)	
H40A	0.017872	0.791796	0.551371	0.057*	
H40B	0.077751	0.799017	0.547017	0.057*	
C41	0.0408 (7)	0.9406 (15)	0.4498 (9)	0.046 (4)	
H41A	0.010402	0.977684	0.470652	0.055*	
H41B	0.070461	0.986418	0.468538	0.055*	
C42	0.0390 (9)	0.961 (2)	0.3828 (10)	0.073 (6)	
H42A	0.036900	1.058186	0.373676	0.110*	
H42B	0.069346	0.923976	0.362836	0.110*	
H42C	0.009558	0.915281	0.364940	0.110*	
C43	0.0841 (11)	0.614 (3)	0.3205 (11)	0.087 (9)	
H43A	0.065395	0.699277	0.321833	0.131*	
H43B	0.085209	0.574427	0.364004	0.131*	
H43C	0.118219	0.631189	0.305314	0.131*	
C44	0.0579 (8)	0.515 (2)	0.2740 (9)	0.057 (5)	
H44A	0.023291	0.498075	0.288902	0.068*	
H44B	0.056384	0.554979	0.230065	0.068*	
C45	0.0627 (8)	0.298 (2)	0.2275 (8)	0.051 (4)	
H45A	0.025870	0.299040	0.232993	0.062*	
H45B	0.074628	0.206030	0.238549	0.062*	
C46	0.0748 (9)	0.326 (2)	0.1574 (9)	0.069 (6)	
H46A	0.058863	0.257949	0.129959	0.104*	
H46B	0.062365	0.416238	0.145643	0.104*	
H46C	0.111179	0.323123	0.151205	0.104*	
C47	0.0695 (9)	−0.089 (2)	0.6989 (9)	0.060 (6)	
H47A	0.056533	−0.174994	0.715149	0.090*	
H47B	0.055627	−0.070924	0.655783	0.090*	
H47C	0.106089	−0.093155	0.696159	0.090*	
C48	0.0544 (8)	0.024 (2)	0.7447 (8)	0.052 (4)	
H48A	0.017518	0.030157	0.747938	0.062*	
H48B	0.068217	0.007822	0.788502	0.062*	
C49	0.0599 (7)	0.2660 (18)	0.7536 (10)	0.053 (4)	
H49A	0.081330	0.276570	0.792397	0.063*	
H49B	0.024761	0.256431	0.767869	0.063*	
C50	0.0654 (7)	0.3909 (17)	0.7089 (9)	0.049 (4)	
H50A	0.055352	0.472484	0.732478	0.074*	
H50B	0.100400	0.399676	0.695133	0.074*	
H50C	0.044044	0.379613	0.670697	0.074*	

### Atomic displacement parameters (Å^2^)

**Table:**

	*U*^11^	*U*^22^	*U*^33^	*U*^12^	*U*^13^	*U*^23^
Ru1	0.0292 (4)	0.0228 (4)	0.0298 (4)	−0.0012 (5)	−0.0013 (8)	−0.0070 (5)
P1	0.047 (3)	0.039 (2)	0.031 (2)	0.001 (2)	0.004 (2)	−0.0052 (18)
P2	0.039 (3)	0.050 (3)	0.043 (2)	−0.001 (2)	−0.002 (2)	−0.013 (2)
F1	0.055 (6)	0.054 (6)	0.070 (7)	−0.001 (5)	0.019 (5)	0.005 (5)
F2	0.079 (8)	0.071 (8)	0.074 (8)	−0.012 (7)	0.038 (6)	−0.004 (6)
F3	0.066 (7)	0.072 (8)	0.054 (6)	0.000 (6)	−0.023 (5)	0.000 (5)
F4	0.102 (9)	0.089 (9)	0.053 (7)	−0.024 (8)	−0.027 (6)	0.022 (6)
F5	0.088 (9)	0.048 (6)	0.052 (6)	−0.007 (6)	0.014 (6)	−0.022 (5)
F6	0.090 (9)	0.041 (5)	0.048 (6)	0.000 (5)	0.020 (6)	−0.012 (5)
F7	0.082 (9)	0.066 (8)	0.150 (12)	−0.008 (7)	−0.063 (9)	0.011 (8)
F8	0.074 (8)	0.149 (12)	0.069 (8)	0.001 (8)	−0.026 (7)	0.035 (8)
F9	0.080 (8)	0.073 (8)	0.068 (7)	−0.013 (7)	0.013 (6)	0.003 (6)
F10	0.078 (8)	0.067 (8)	0.120 (10)	0.003 (7)	0.051 (8)	−0.001 (8)
F11	0.084 (9)	0.080 (7)	0.078 (8)	−0.019 (7)	0.012 (7)	−0.042 (7)
F12	0.088 (10)	0.050 (6)	0.112 (10)	−0.002 (6)	0.022 (8)	−0.030 (7)
O1	0.061 (7)	0.028 (5)	0.040 (6)	0.004 (5)	−0.003 (5)	0.003 (5)
O2	0.051 (8)	0.055 (7)	0.037 (6)	−0.003 (6)	−0.016 (5)	−0.004 (5)
O3	0.038 (6)	0.042 (6)	0.046 (7)	0.002 (5)	0.010 (5)	−0.014 (5)
N1	0.034 (4)	0.021 (4)	0.028 (4)	−0.006 (4)	0.003 (3)	0.000 (3)
N2	0.030 (4)	0.019 (4)	0.028 (4)	0.002 (4)	−0.002 (3)	0.002 (3)
N3	0.028 (3)	0.012 (3)	0.028 (3)	−0.004 (3)	−0.001 (4)	0.002 (3)
N4	0.029 (4)	0.013 (4)	0.021 (4)	−0.003 (3)	0.001 (3)	−0.002 (3)
N5	0.033 (4)	0.030 (5)	0.029 (4)	0.001 (4)	0.005 (3)	0.004 (4)
N6	0.030 (4)	0.020 (4)	0.010 (4)	0.005 (3)	−0.005 (3)	0.007 (3)
N7	0.034 (4)	0.030 (4)	0.042 (5)	0.000 (4)	−0.002 (4)	−0.008 (4)
N8	0.030 (3)	0.029 (3)	0.030 (4)	0.002 (3)	−0.001 (4)	−0.006 (3)
N9	0.034 (4)	0.028 (4)	0.024 (4)	0.000 (3)	0.001 (4)	−0.002 (4)
N10	0.035 (4)	0.031 (4)	0.032 (5)	0.002 (4)	−0.002 (4)	−0.005 (4)
C1	0.028 (4)	0.009 (4)	0.011 (4)	0.002 (3)	−0.001 (3)	0.002 (3)
C2	0.030 (4)	0.025 (5)	0.024 (4)	−0.001 (4)	0.001 (4)	−0.003 (4)
C3	0.037 (5)	0.038 (5)	0.035 (5)	0.001 (4)	0.006 (4)	0.000 (4)
C4	0.048 (5)	0.050 (6)	0.034 (5)	−0.001 (4)	0.005 (4)	0.002 (4)
C5	0.042 (5)	0.036 (5)	0.032 (5)	0.002 (4)	−0.001 (4)	0.002 (4)
C6	0.028 (4)	0.021 (5)	0.027 (4)	0.002 (4)	−0.003 (3)	−0.003 (4)
C7	0.034 (4)	0.026 (5)	0.027 (4)	−0.003 (4)	0.000 (4)	0.000 (4)
C8	0.034 (4)	0.016 (4)	0.028 (4)	−0.001 (4)	0.000 (4)	0.002 (4)
C9	0.036 (4)	0.029 (5)	0.030 (5)	0.000 (4)	−0.002 (4)	0.001 (4)
C10	0.035 (4)	0.035 (4)	0.036 (4)	0.003 (4)	0.000 (4)	0.005 (4)
C11	0.032 (4)	0.019 (5)	0.025 (4)	0.000 (4)	0.004 (4)	−0.001 (4)
C12	0.030 (4)	0.016 (4)	0.024 (4)	0.001 (4)	0.000 (3)	−0.001 (4)
C13	0.031 (4)	0.021 (5)	0.023 (4)	−0.001 (4)	−0.001 (3)	−0.004 (4)
C14	0.032 (4)	0.030 (5)	0.031 (4)	−0.002 (4)	0.003 (4)	−0.002 (4)
C15	0.046 (5)	0.045 (6)	0.038 (5)	0.001 (4)	−0.002 (4)	−0.003 (4)
C16	0.042 (5)	0.036 (5)	0.034 (5)	0.003 (4)	−0.004 (4)	0.001 (4)
C17	0.038 (5)	0.035 (5)	0.033 (5)	0.004 (4)	−0.001 (4)	0.000 (4)
C18	0.037 (5)	0.033 (5)	0.033 (5)	−0.001 (4)	0.000 (4)	−0.001 (4)
C19	0.036 (4)	0.024 (5)	0.028 (5)	−0.001 (4)	−0.001 (4)	0.007 (4)
C20	0.029 (4)	0.017 (4)	0.021 (5)	0.001 (3)	−0.001 (4)	0.004 (4)
C21	0.032 (4)	0.022 (4)	0.022 (5)	0.000 (4)	0.002 (4)	0.006 (4)
C22	0.038 (5)	0.035 (5)	0.036 (5)	0.004 (4)	−0.003 (4)	−0.001 (4)
C23	0.044 (5)	0.031 (5)	0.032 (5)	0.003 (4)	−0.002 (4)	−0.003 (4)
C24	0.041 (5)	0.027 (5)	0.031 (5)	0.002 (4)	−0.005 (4)	−0.001 (4)
C25	0.034 (5)	0.029 (4)	0.025 (5)	0.002 (4)	−0.003 (4)	0.000 (4)
C26	0.030 (4)	0.026 (4)	0.021 (4)	−0.001 (3)	0.001 (4)	−0.001 (4)
C27	0.029 (4)	0.035 (4)	0.025 (5)	0.000 (3)	−0.002 (4)	−0.001 (4)
C28	0.031 (4)	0.033 (4)	0.027 (5)	−0.002 (4)	−0.001 (4)	0.000 (4)
C29	0.033 (5)	0.036 (5)	0.036 (5)	0.001 (4)	0.000 (4)	0.000 (4)
C30	0.034 (4)	0.030 (4)	0.026 (5)	0.002 (4)	−0.001 (4)	0.003 (4)
C31	0.032 (4)	0.027 (4)	0.024 (5)	0.001 (3)	−0.004 (4)	−0.005 (4)
C32	0.034 (4)	0.029 (4)	0.024 (5)	0.000 (4)	−0.001 (4)	−0.004 (4)
C33	0.033 (4)	0.025 (4)	0.021 (5)	0.002 (3)	0.001 (4)	−0.003 (4)
C34	0.036 (5)	0.020 (4)	0.024 (5)	0.005 (4)	0.000 (4)	0.005 (4)
C35	0.041 (5)	0.033 (5)	0.034 (5)	−0.002 (4)	0.001 (4)	0.001 (4)
C36	0.038 (5)	0.033 (5)	0.035 (5)	−0.006 (4)	0.003 (4)	−0.001 (4)
C37	0.031 (4)	0.023 (4)	0.018 (4)	0.003 (4)	−0.003 (4)	0.003 (4)
C38	0.033 (4)	0.020 (4)	0.018 (4)	0.000 (3)	0.003 (4)	0.005 (4)
C39	0.101 (18)	0.049 (10)	0.075 (14)	−0.004 (11)	−0.028 (13)	0.031 (10)
C40	0.054 (10)	0.049 (9)	0.039 (8)	0.005 (8)	−0.004 (7)	−0.002 (7)
C41	0.061 (11)	0.019 (6)	0.059 (9)	0.003 (7)	−0.005 (8)	−0.012 (6)
C42	0.113 (19)	0.053 (12)	0.054 (9)	0.014 (12)	−0.002 (11)	0.001 (9)
C43	0.11 (2)	0.085 (15)	0.064 (15)	0.028 (14)	−0.053 (14)	−0.038 (13)
C44	0.061 (12)	0.066 (10)	0.043 (11)	0.010 (9)	−0.020 (9)	−0.017 (8)
C45	0.066 (12)	0.048 (9)	0.040 (8)	−0.011 (9)	−0.013 (8)	0.006 (7)
C46	0.112 (18)	0.052 (12)	0.043 (9)	0.024 (12)	0.002 (10)	0.002 (8)
C47	0.096 (17)	0.051 (9)	0.031 (9)	−0.032 (10)	0.022 (10)	0.003 (7)
C48	0.060 (12)	0.068 (9)	0.027 (9)	−0.003 (9)	0.009 (8)	0.001 (7)
C49	0.056 (10)	0.054 (8)	0.048 (9)	0.018 (8)	0.013 (9)	−0.012 (7)
C50	0.050 (10)	0.036 (8)	0.061 (11)	0.004 (7)	−0.012 (8)	−0.017 (7)

### Geometric parameters (Å, º)

**Table:**

Ru1—N8	1.983 (9)	C16—C17	1.35 (2)
Ru1—N3	2.011 (8)	C16—H16	0.9500
Ru1—N1	2.046 (13)	C17—C18	1.39 (2)
Ru1—N6	2.053 (12)	C17—H17	0.9500
Ru1—N9	2.094 (13)	C18—C19	1.41 (2)
Ru1—N4	2.104 (12)	C18—H18	0.9500
P1—F5	1.570 (12)	C20—C25	1.41 (2)
P1—F4	1.571 (14)	C20—C21	1.46 (2)
P1—F3	1.586 (12)	C21—C22	1.35 (2)
P1—F2	1.591 (13)	C21—H21	0.9500
P1—F1	1.597 (12)	C22—C23	1.41 (2)
P1—F6	1.607 (12)	C22—H22	0.9500
P2—F7	1.525 (15)	C23—C24	1.37 (2)
P2—F10	1.569 (15)	C23—H23	0.9500
P2—F12	1.575 (14)	C24—C25	1.44 (2)
P2—F9	1.586 (14)	C24—H24	0.9500
P2—F8	1.607 (14)	C26—C27	1.48 (2)
P2—F11	1.615 (13)	C27—C28	1.386 (19)
O1—C40	1.44 (2)	C28—C29	1.385 (19)
O1—C41	1.472 (19)	C28—H28	0.9500
O2—C44	1.40 (2)	C29—C30	1.40 (2)
O2—C45	1.44 (2)	C29—H29	0.9500
O3—C49	1.419 (19)	C30—C31	1.38 (2)
O3—C48	1.47 (2)	C30—H30	0.9500
N1—C7	1.34 (2)	C31—C32	1.43 (2)
N1—C1	1.377 (17)	C33—C34	1.38 (2)
N2—C7	1.340 (19)	C33—C38	1.41 (2)
N2—C6	1.42 (2)	C34—C35	1.37 (2)
N2—H2N	0.90 (3)	C34—H34	0.9500
N3—C12	1.32 (2)	C35—C36	1.43 (2)
N3—C8	1.39 (2)	C35—H35	0.9500
N4—C13	1.36 (2)	C36—C37	1.39 (2)
N4—C19	1.449 (19)	C36—H36	0.9500
N5—C14	1.34 (2)	C37—C38	1.34 (2)
N5—C13	1.402 (19)	C37—H37	0.9500
N5—H5N	0.91 (3)	C39—C40	1.50 (3)
N6—C26	1.36 (2)	C39—H39A	0.9800
N6—C20	1.392 (18)	C39—H39B	0.9800
N7—C26	1.321 (19)	C39—H39C	0.9800
N7—C25	1.42 (2)	C40—H40A	0.9900
N7—H7N	0.90 (3)	C40—H40B	0.9900
N8—C31	1.330 (19)	C41—C42	1.40 (3)
N8—C27	1.432 (18)	C41—H41A	0.9900
N9—C32	1.32 (2)	C41—H41B	0.9900
N9—C38	1.380 (19)	C42—H42A	0.9800
N10—C33	1.39 (2)	C42—H42B	0.9800
N10—C32	1.396 (19)	C42—H42C	0.9800
N10—H10N	0.89 (3)	C43—C44	1.54 (3)
C1—C2	1.38 (2)	C43—H43A	0.9800
C1—C6	1.435 (19)	C43—H43B	0.9800
C2—C3	1.38 (2)	C43—H43C	0.9800
C2—H2	0.9500	C44—H44A	0.9900
C3—C4	1.45 (3)	C44—H44B	0.9900
C3—H3	0.9500	C45—C46	1.51 (2)
C4—C5	1.36 (3)	C45—H45A	0.9900
C4—H4	0.9500	C45—H45B	0.9900
C5—C6	1.41 (2)	C46—H46A	0.9800
C5—H5	0.9500	C46—H46B	0.9800
C7—C8	1.49 (2)	C46—H46C	0.9800
C8—C9	1.36 (2)	C47—C48	1.52 (3)
C9—C10	1.40 (3)	C47—H47A	0.9800
C9—H9	0.9500	C47—H47B	0.9800
C10—C11	1.38 (3)	C47—H47C	0.9800
C10—H10	0.9500	C48—H48A	0.9900
C11—C12	1.41 (2)	C48—H48B	0.9900
C11—H11	0.9500	C49—C50	1.55 (3)
C12—C13	1.43 (2)	C49—H49A	0.9900
C14—C19	1.38 (2)	C49—H49B	0.9900
C14—C15	1.42 (2)	C50—H50A	0.9800
C15—C16	1.39 (3)	C50—H50B	0.9800
C15—H15	0.9500	C50—H50C	0.9800
			
N8—Ru1—N3	177.3 (7)	C14—C19—C18	126.6 (15)
N8—Ru1—N1	99.1 (7)	C14—C19—N4	107.5 (14)
N3—Ru1—N1	79.5 (6)	C18—C19—N4	125.9 (15)
N8—Ru1—N6	78.9 (5)	N6—C20—C25	111.4 (13)
N3—Ru1—N6	103.5 (5)	N6—C20—C21	129.0 (13)
N1—Ru1—N6	95.0 (5)	C25—C20—C21	119.6 (13)
N8—Ru1—N9	77.3 (6)	C22—C21—C20	116.2 (14)
N3—Ru1—N9	100.3 (5)	C22—C21—H21	121.9
N1—Ru1—N9	91.0 (5)	C20—C21—H21	121.9
N6—Ru1—N9	156.1 (3)	C21—C22—C23	123.5 (16)
N8—Ru1—N4	104.5 (6)	C21—C22—H22	118.2
N3—Ru1—N4	76.9 (6)	C23—C22—H22	118.2
N1—Ru1—N4	156.4 (4)	C24—C23—C22	123.1 (16)
N6—Ru1—N4	91.5 (4)	C24—C23—H23	118.5
N9—Ru1—N4	92.1 (5)	C22—C23—H23	118.5
F5—P1—F4	90.2 (8)	C23—C24—C25	115.0 (16)
F5—P1—F3	90.1 (7)	C23—C24—H24	122.5
F4—P1—F3	179.6 (10)	C25—C24—H24	122.5
F5—P1—F2	91.7 (8)	C20—C25—N7	104.4 (13)
F4—P1—F2	90.8 (9)	C20—C25—C24	122.6 (15)
F3—P1—F2	89.5 (8)	N7—C25—C24	133.0 (15)
F5—P1—F1	89.2 (7)	N7—C26—N6	115.8 (14)
F4—P1—F1	89.1 (8)	N7—C26—C27	127.8 (14)
F3—P1—F1	90.6 (7)	N6—C26—C27	115.9 (12)
F2—P1—F1	179.1 (8)	C28—C27—N8	121.8 (12)
F5—P1—F6	178.3 (7)	C28—C27—C26	128.3 (13)
F4—P1—F6	90.4 (8)	N8—C27—C26	109.9 (12)
F3—P1—F6	89.3 (7)	C29—C28—C27	118.2 (13)
F2—P1—F6	89.9 (7)	C29—C28—H28	120.9
F1—P1—F6	89.1 (6)	C27—C28—H28	120.9
F7—P2—F10	88.7 (10)	C28—C29—C30	119.8 (13)
F7—P2—F12	91.9 (9)	C28—C29—H29	120.1
F10—P2—F12	89.5 (8)	C30—C29—H29	120.1
F7—P2—F9	92.3 (10)	C31—C30—C29	120.0 (14)
F10—P2—F9	179.0 (9)	C31—C30—H30	120.0
F12—P2—F9	90.8 (8)	C29—C30—H30	120.0
F7—P2—F8	177.9 (10)	N8—C31—C30	122.4 (13)
F10—P2—F8	92.1 (9)	N8—C31—C32	106.3 (13)
F12—P2—F8	90.1 (10)	C30—C31—C32	131.2 (14)
F9—P2—F8	86.9 (8)	N9—C32—N10	106.7 (14)
F7—P2—F11	91.2 (9)	N9—C32—C31	123.7 (14)
F10—P2—F11	91.7 (8)	N10—C32—C31	129.6 (15)
F12—P2—F11	176.6 (9)	C34—C33—N10	132.5 (14)
F9—P2—F11	87.9 (7)	C34—C33—C38	122.2 (15)
F8—P2—F11	86.7 (9)	N10—C33—C38	105.3 (13)
C40—O1—C41	113.2 (13)	C35—C34—C33	114.5 (15)
C44—O2—C45	111.9 (14)	C35—C34—H34	122.7
C49—O3—C48	112.5 (13)	C33—C34—H34	122.7
C7—N1—C1	106.9 (13)	C34—C35—C36	125.3 (16)
C7—N1—Ru1	113.7 (10)	C34—C35—H35	117.3
C1—N1—Ru1	139.2 (11)	C36—C35—H35	117.3
C7—N2—C6	108.2 (13)	C37—C36—C35	116.4 (15)
C7—N2—H2N	125 (10)	C37—C36—H36	121.8
C6—N2—H2N	127 (10)	C35—C36—H36	121.8
C12—N3—C8	119.1 (9)	C38—C37—C36	119.5 (14)
C12—N3—Ru1	121.7 (12)	C38—C37—H37	120.2
C8—N3—Ru1	118.5 (11)	C36—C37—H37	120.2
C13—N4—C19	104.5 (12)	C37—C38—N9	131.0 (14)
C13—N4—Ru1	110.7 (10)	C37—C38—C33	121.8 (14)
C19—N4—Ru1	144.8 (11)	N9—C38—C33	107.2 (13)
C14—N5—C13	107.2 (14)	C40—C39—H39A	109.5
C14—N5—H5N	127 (10)	C40—C39—H39B	109.5
C13—N5—H5N	126 (10)	H39A—C39—H39B	109.5
C26—N6—C20	101.9 (12)	C40—C39—H39C	109.5
C26—N6—Ru1	116.0 (9)	H39A—C39—H39C	109.5
C20—N6—Ru1	142.1 (11)	H39B—C39—H39C	109.5
C26—N7—C25	106.2 (13)	O1—C40—C39	109.7 (15)
C26—N7—H7N	132 (3)	O1—C40—H40A	109.7
C25—N7—H7N	122 (3)	C39—C40—H40A	109.7
C31—N8—C27	117.5 (10)	O1—C40—H40B	109.7
C31—N8—Ru1	122.6 (10)	C39—C40—H40B	109.7
C27—N8—Ru1	118.8 (9)	H40A—C40—H40B	108.2
C32—N9—C38	111.1 (13)	C42—C41—O1	107.0 (14)
C32—N9—Ru1	109.4 (10)	C42—C41—H41A	110.3
C38—N9—Ru1	139.0 (11)	O1—C41—H41A	110.3
C33—N10—C32	109.5 (13)	C42—C41—H41B	110.3
C33—N10—H10N	126 (2)	O1—C41—H41B	110.3
C32—N10—H10N	125 (3)	H41A—C41—H41B	108.6
N1—C1—C2	133.6 (13)	C41—C42—H42A	109.5
N1—C1—C6	109.0 (13)	C41—C42—H42B	109.5
C2—C1—C6	117.0 (12)	H42A—C42—H42B	109.5
C3—C2—C1	119.3 (15)	C41—C42—H42C	109.5
C3—C2—H2	120.3	H42A—C42—H42C	109.5
C1—C2—H2	120.3	H42B—C42—H42C	109.5
C2—C3—C4	120.7 (17)	C44—C43—H43A	109.5
C2—C3—H3	119.6	C44—C43—H43B	109.5
C4—C3—H3	119.6	H43A—C43—H43B	109.5
C5—C4—C3	123.4 (18)	C44—C43—H43C	109.5
C5—C4—H4	118.3	H43A—C43—H43C	109.5
C3—C4—H4	118.3	H43B—C43—H43C	109.5
C4—C5—C6	113.0 (17)	O2—C44—C43	109.4 (16)
C4—C5—H5	123.5	O2—C44—H44A	109.8
C6—C5—H5	123.5	C43—C44—H44A	109.8
C5—C6—N2	129.8 (15)	O2—C44—H44B	109.8
C5—C6—C1	126.5 (16)	C43—C44—H44B	109.8
N2—C6—C1	103.6 (12)	H44A—C44—H44B	108.2
N1—C7—N2	112.1 (14)	O2—C45—C46	113.8 (16)
N1—C7—C8	119.1 (14)	O2—C45—H45A	108.8
N2—C7—C8	128.7 (16)	C46—C45—H45A	108.8
C9—C8—N3	123.6 (14)	O2—C45—H45B	108.8
C9—C8—C7	127.4 (15)	C46—C45—H45B	108.8
N3—C8—C7	109.0 (14)	H45A—C45—H45B	107.7
C8—C9—C10	116.5 (15)	C45—C46—H46A	109.5
C8—C9—H9	121.7	C45—C46—H46B	109.5
C10—C9—H9	121.7	H46A—C46—H46B	109.5
C11—C10—C9	120.5 (10)	C45—C46—H46C	109.5
C11—C10—H10	119.8	H46A—C46—H46C	109.5
C9—C10—H10	119.8	H46B—C46—H46C	109.5
C10—C11—C12	119.5 (14)	C48—C47—H47A	109.5
C10—C11—H11	120.2	C48—C47—H47B	109.5
C12—C11—H11	120.2	H47A—C47—H47B	109.5
N3—C12—C11	120.4 (14)	C48—C47—H47C	109.5
N3—C12—C13	109.5 (14)	H47A—C47—H47C	109.5
C11—C12—C13	129.9 (15)	H47B—C47—H47C	109.5
N4—C13—N5	110.8 (14)	O3—C48—C47	106.8 (13)
N4—C13—C12	120.5 (14)	O3—C48—H48A	110.4
N5—C13—C12	128.7 (15)	C47—C48—H48A	110.4
N5—C14—C19	110.0 (15)	O3—C48—H48B	110.4
N5—C14—C15	134.5 (16)	C47—C48—H48B	110.4
C19—C14—C15	115.5 (17)	H48A—C48—H48B	108.6
C16—C15—C14	118.1 (18)	O3—C49—C50	107.9 (15)
C16—C15—H15	121.0	O3—C49—H49A	110.1
C14—C15—H15	121.0	C50—C49—H49A	110.1
C17—C16—C15	124.2 (18)	O3—C49—H49B	110.1
C17—C16—H16	117.9	C50—C49—H49B	110.1
C15—C16—H16	117.9	H49A—C49—H49B	108.4
C16—C17—C18	120.6 (18)	C49—C50—H50A	109.5
C16—C17—H17	119.7	C49—C50—H50B	109.5
C18—C17—H17	119.7	H50A—C50—H50B	109.5
C17—C18—C19	114.7 (16)	C49—C50—H50C	109.5
C17—C18—H18	122.6	H50A—C50—H50C	109.5
C19—C18—H18	122.6	H50B—C50—H50C	109.5
			
C7—N1—C1—C2	170.7 (15)	Ru1—N6—C20—C21	−3 (2)
Ru1—N1—C1—C2	−4 (3)	N6—C20—C21—C22	179.8 (14)
C7—N1—C1—C6	−1.8 (16)	C25—C20—C21—C22	1 (2)
Ru1—N1—C1—C6	−176.8 (11)	C20—C21—C22—C23	−3 (2)
N1—C1—C2—C3	−174.8 (15)	C21—C22—C23—C24	1 (2)
C6—C1—C2—C3	−3 (2)	C22—C23—C24—C25	2 (2)
C1—C2—C3—C4	1 (2)	N6—C20—C25—N7	1.8 (17)
C2—C3—C4—C5	0 (3)	C21—C20—C25—N7	−179.5 (13)
C3—C4—C5—C6	−1 (3)	N6—C20—C25—C24	−176.6 (13)
C4—C5—C6—N2	174.9 (16)	C21—C20—C25—C24	2 (2)
C4—C5—C6—C1	−1 (2)	C26—N7—C25—C20	1.0 (17)
C7—N2—C6—C5	−174.4 (16)	C26—N7—C25—C24	179.2 (17)
C7—N2—C6—C1	1.9 (15)	C23—C24—C25—C20	−4 (2)
N1—C1—C6—C5	176.4 (14)	C23—C24—C25—N7	178.2 (17)
C2—C1—C6—C5	3 (2)	C25—N7—C26—N6	−3.7 (19)
N1—C1—C6—N2	−0.1 (14)	C25—N7—C26—C27	−175.4 (15)
C2—C1—C6—N2	−174.0 (12)	C20—N6—C26—N7	4.6 (17)
C1—N1—C7—N2	3.2 (17)	Ru1—N6—C26—N7	−175.0 (10)
Ru1—N1—C7—N2	179.6 (10)	C20—N6—C26—C27	177.3 (12)
C1—N1—C7—C8	−173.6 (13)	Ru1—N6—C26—C27	−2.3 (16)
Ru1—N1—C7—C8	2.9 (18)	C31—N8—C27—C28	−2 (2)
C6—N2—C7—N1	−3.3 (18)	Ru1—N8—C27—C28	−170.4 (12)
C6—N2—C7—C8	173.1 (15)	C31—N8—C27—C26	175.9 (14)
C12—N3—C8—C9	−6 (3)	Ru1—N8—C27—C26	7.5 (19)
Ru1—N3—C8—C9	−176.8 (13)	N7—C26—C27—C28	−14 (3)
C12—N3—C8—C7	174.9 (11)	N6—C26—C27—C28	174.6 (15)
Ru1—N3—C8—C7	4.3 (16)	N7—C26—C27—N8	168.6 (16)
N1—C7—C8—C9	176.6 (16)	N6—C26—C27—N8	−3.1 (19)
N2—C7—C8—C9	0 (3)	N8—C27—C28—C29	0 (2)
N1—C7—C8—N3	−5 (2)	C26—C27—C28—C29	−177.6 (15)
N2—C7—C8—N3	179.2 (15)	C27—C28—C29—C30	0 (2)
N3—C8—C9—C10	3 (3)	C28—C29—C30—C31	3 (2)
C7—C8—C9—C10	−178.2 (16)	C27—N8—C31—C30	5 (2)
C8—C9—C10—C11	−1 (3)	Ru1—N8—C31—C30	172.6 (11)
C9—C10—C11—C12	3 (3)	C27—N8—C31—C32	−175.9 (14)
C8—N3—C12—C11	7 (2)	Ru1—N8—C31—C32	−8.0 (19)
Ru1—N3—C12—C11	177.5 (11)	C29—C30—C31—N8	−5 (2)
C8—N3—C12—C13	−177.8 (11)	C29—C30—C31—C32	175.5 (15)
Ru1—N3—C12—C13	−7.5 (17)	C38—N9—C32—N10	−4.2 (18)
C10—C11—C12—N3	−6 (2)	Ru1—N9—C32—N10	−177.8 (10)
C10—C11—C12—C13	−179.5 (16)	C38—N9—C32—C31	175.8 (14)
C19—N4—C13—N5	1.4 (15)	Ru1—N9—C32—C31	2.3 (19)
Ru1—N4—C13—N5	−177.6 (9)	C33—N10—C32—N9	4.3 (18)
C19—N4—C13—C12	−175.6 (13)	C33—N10—C32—C31	−175.8 (15)
Ru1—N4—C13—C12	5.4 (17)	N8—C31—C32—N9	3 (2)
C14—N5—C13—N4	−0.9 (17)	C30—C31—C32—N9	−177.4 (15)
C14—N5—C13—C12	175.8 (15)	N8—C31—C32—N10	−176.7 (16)
N3—C12—C13—N4	1 (2)	C30—C31—C32—N10	3 (3)
C11—C12—C13—N4	175.2 (15)	C32—N10—C33—C34	177.4 (16)
N3—C12—C13—N5	−175.7 (14)	C32—N10—C33—C38	−2.7 (17)
C11—C12—C13—N5	−1 (3)	N10—C33—C34—C35	−177.1 (16)
C13—N5—C14—C19	0.0 (18)	C38—C33—C34—C35	3 (2)
C13—N5—C14—C15	−177.4 (18)	C33—C34—C35—C36	−2 (2)
N5—C14—C15—C16	175.0 (18)	C34—C35—C36—C37	0 (2)
C19—C14—C15—C16	−2 (2)	C35—C36—C37—C38	3 (2)
C14—C15—C16—C17	3 (3)	C36—C37—C38—N9	176.7 (14)
C15—C16—C17—C18	0 (3)	C36—C37—C38—C33	−2 (2)
C16—C17—C18—C19	−3 (2)	C32—N9—C38—C37	−176.3 (15)
N5—C14—C19—C18	−179.3 (16)	Ru1—N9—C38—C37	−6 (3)
C15—C14—C19—C18	−1 (3)	C32—N9—C38—C33	2.6 (17)
N5—C14—C19—N4	0.9 (18)	Ru1—N9—C38—C33	173.4 (12)
C15—C14—C19—N4	178.9 (13)	C34—C33—C38—C37	−1 (2)
C17—C18—C19—C14	4 (2)	N10—C33—C38—C37	179.2 (14)
C17—C18—C19—N4	−176.0 (14)	C34—C33—C38—N9	−180.0 (14)
C13—N4—C19—C14	−1.4 (16)	N10—C33—C38—N9	0.1 (16)
Ru1—N4—C19—C14	176.9 (12)	C41—O1—C40—C39	179.7 (15)
C13—N4—C19—C18	178.8 (15)	C40—O1—C41—C42	178.4 (18)
Ru1—N4—C19—C18	−3 (3)	C45—O2—C44—C43	−178.1 (18)
C26—N6—C20—C25	−3.7 (15)	C44—O2—C45—C46	78 (2)
Ru1—N6—C20—C25	175.7 (11)	C49—O3—C48—C47	175.3 (17)
C26—N6—C20—C21	177.7 (14)	C48—O3—C49—C50	−158.7 (15)

### Hydrogen-bond geometry (Å, º)

**Table:**

*D*—H···*A*	*D*—H	H···*A*	*D*···*A*	*D*—H···*A*
N2—H2*N*···O2	0.90 (3)	1.85 (5)	2.730 (18)	166 (17)
N5—H5*N*···O3	0.91 (3)	1.81 (5)	2.704 (19)	170 (18)
N7—H7*N*···F8	0.90 (3)	2.58 (7)	3.30 (2)	137 (8)
N7—H7*N*···F11	0.90 (3)	2.05 (4)	2.93 (2)	167 (10)
N10—H10*N*···F6^i^	0.89 (3)	2.16 (3)	3.028 (19)	166 (6)

Symmetry code: (i) *x*, *y*−1, *z*.
